# Supplementary material for: Obesity Is Associated with Increased F2-Isoprostanes and IL-6 in Black Women
Source: Endocrines. Author manuscript; Available in PMC 2026 Mar 7. (PMC12965753; doi:10.3390/endocrines4010003)
Supplement: Supplementary material [file NIHMS2144190-supplement-Supplementary_material.pdf]

**Supplementary Materials:** The following supporting information can be downloaded at: https://www.mdpi.com/article/10.3390/endocrines4010003/s1,

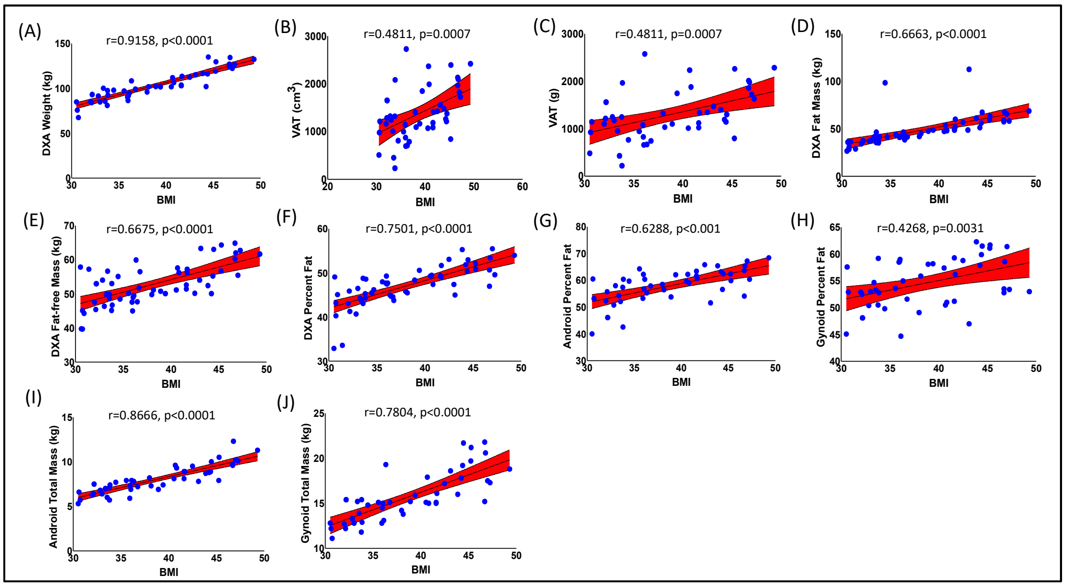


**Figure S1. Relationship between BMI and anthropometric DXA measurements.** Our data show very strong positive correlation between BMI and the following variables: (A) DXA weight, (r=0.9158, p=<0.0001), (B) VAT (cm$^3$, r=0.4811, p=0.0007), (C) e-VAT (g, r=0.4810, p=0.0007), (D) DXA fat mass (r=0.6663, p=<0.0001), (E) DXA fat free mass (r=0.6675, p=<0.0001), (F) DXA percent fat (r=0.7501, p=<0.0001), (G) android percent fat (r=0.6288, p=<0.0001), (H) gynoid percent fat (r=0.4268, p=<0.0031), (I) android total mass (kg, r=0.5624, p=<0.0001), and (J) gynoid total mass (kg, r=0.7804, p=<0.0001). Pearson's correlation analysis was employed to determine the correlation. P<0.05 was considered statistically significant correlation.

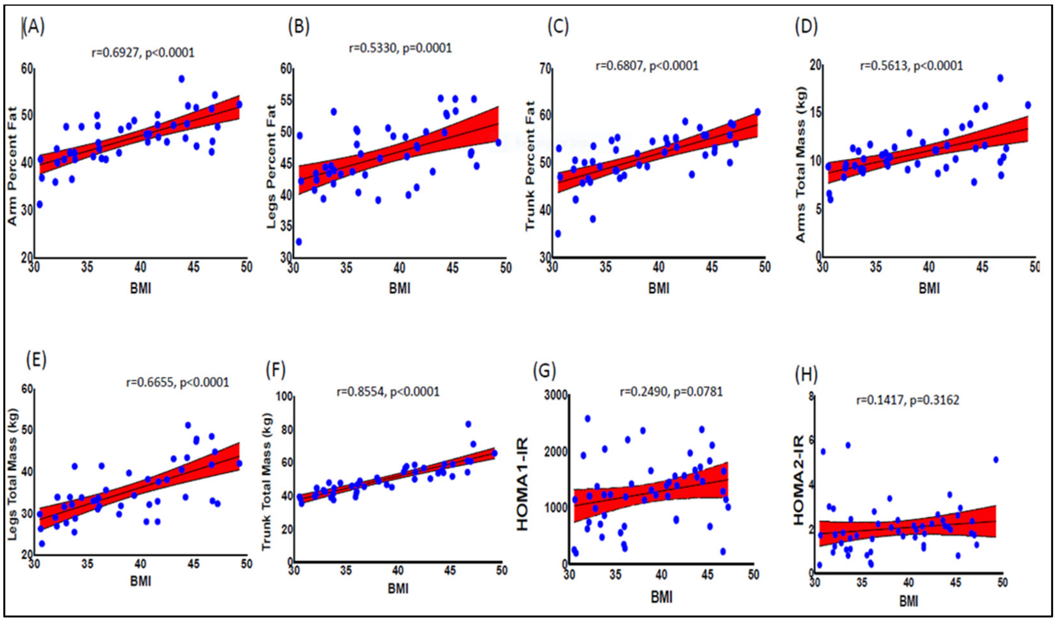


**Figure S2. Relationship of various body parts and their fat content with BMI.** There was strong positive correlation between BMI and (A) arms percent fat (r=0.6927, p=<0.0001), (B) legs percent fat (r=0.5330, p=<0.0001), and (C) trunk (r=0.6807, p=<0.0001). Similarly, there were strong positive correlation between BMI and (D) arms total mass (kg, r=0.5613, p=<0.0001), (E) legs total mass (kg, r=0.6655, p=<0.0001), and (F) trunk total mass (kg, r=0.8550, p=<0.0001), (G) HOMA1-IR (r=0.2490, p=<0.0781), (H) HOMA2-IR (r=0.1417, p=<0.3162). Pearson's correlation analysis was employed to determine the correlation. P<0.05 was considered a statistically significant correlation.

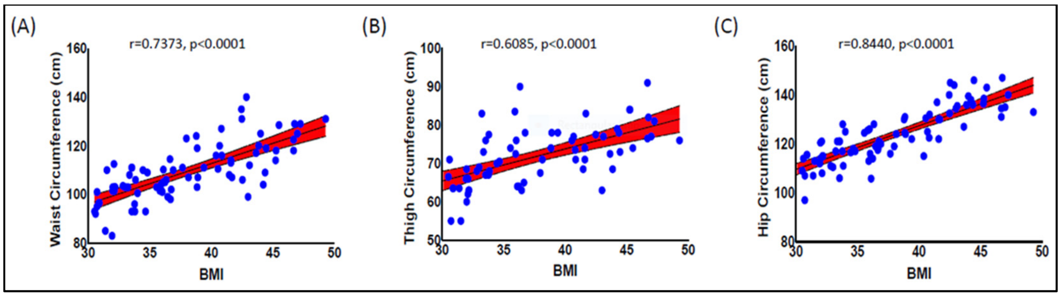


**Figure S3. Relationship between BMI and certain body circumferences.** Our data show strong positive correlation between BMI and circumferences of (A) waist (cm, r=0.7373, p=<0.0001), (B) thigh (cm, r=0.6085, p=<0.0001), and (C) hip (cm, r=0.8401, p=<0.0001). Pearson's correlation analysis was employed to determine the correlation. P<0.05 was considered a statistically significant correlation.

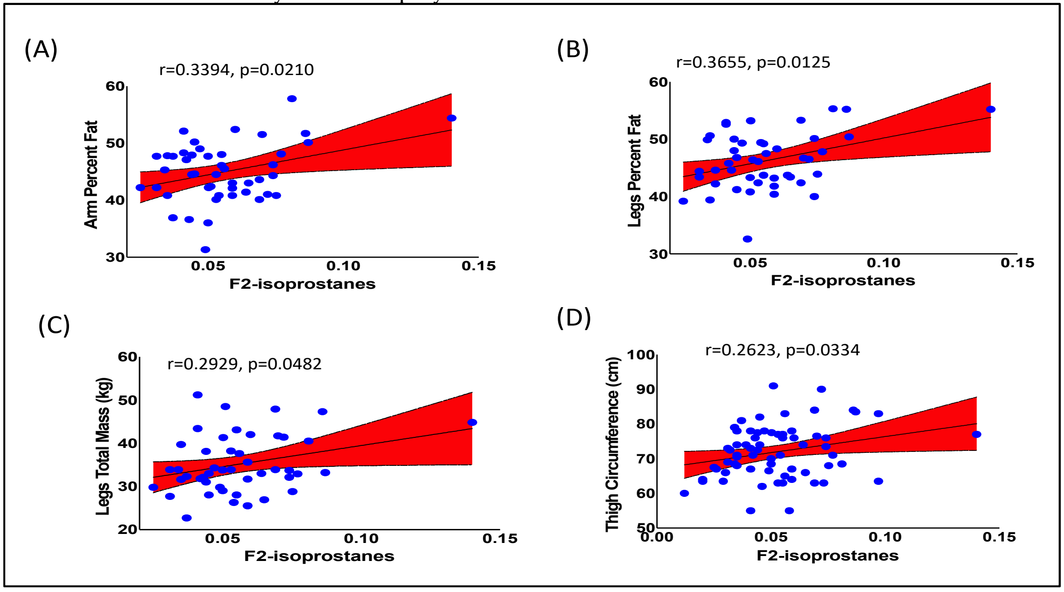


**Figure S4. Relationship between anthropometric DXA measurements and F2-IsoPs.** Our data show a positive correlation between (A) arms percent fat (r=0.3394, p=0.0210), (B) legs percent fat (r=0.3655, p=0.0125), (C) legs total mass (kg, r=0.2929, p=0.0482), and (D) thigh circumferences (cm, r=0.2723, p=0.0256) were correlated with F2-isoprostanes. Pearson's correlation analysis was employed to determine the correlation. P<0.05 was considered a statistically significant correlation.
